# Novel Hepatitis E Virus Genotype in Norway Rats, Germany

**DOI:** 10.3201/eid1609.100444

**Published:** 2010-09

**Authors:** Reimar Johne, Gerald Heckel, Anita Plenge-Bönig, Eveline Kindler, Christina Maresch, Jochen Reetz, Anika Schielke, Rainer G. Ulrich

**Affiliations:** Author affiliations: Federal Institute for Risk Assessment, Berlin, Germany (R. Johne, J. Reetz, A. Schielke);; University of Bern, Bern, Switzerland (G. Heckel, E. Kindler);; Swiss Institute of Bioinformatics, Lausanne, Switzerland (G. Heckel);; Institute of Hygiene and Environment Hamburg, Hamburg, Germany (A. Plenge-Bönig);; Friedrich-Loeffler-Institut, Greifswald–Insel Riems, Germany (C. Maresch, R.G. Ulrich);; Free University of Berlin, Berlin (A. Schielke)

**Keywords:** Hepatitis E virus, Norway rat, complete genome, viruses, dispatch

## Abstract

Human hepatitis E virus infections may be caused by zoonotic transmission of virus genotypes 3 and 4. To determine whether rodents are a reservoir, we analyzed the complete nucleotide sequence of a hepatitis E–like virus from 2 Norway rats in Germany. The sequence suggests a separate genotype for this hepatotropic virus.

Hepatitis E virus (HEV) is a nonenveloped virus, diameter 30–34 nm, that belongs to the genus *Hepevirus*. Its single-stranded, positive-polarity RNA genome of 6.6–7.3 kb harbors 3 major open reading frames (ORFs) flanked by a capped 5′ end and a poly A at the 3′ end. ORF1 at the 5′ end of the genome codes for several nonstructural proteins, ORF2 encodes the immunodominant capsid protein, and the partially overlapping ORF3 codes for a cytoskeleton-associated phosphoprotein with multiple functions (*1*).

Hepatitis E, an acute self-limiting disease, occurs worldwide; large outbreaks have occurred in developing countries, as was recently reported from Uganda (*2*). Initially, hepatitis E was believed to be endemic only to developing countries in Asia, Africa, and Central America, but recent studies have demonstrated autochthonous infections in industrialized countries (Europe, Japan) (*3*). In contrast to the fecal–oral transmission of HEV that occurs in developing countries, it is suspected that these human infections result from zoonotic transmission of HEV genotypes 3 and 4; domestic pigs, wild boars, and deer represent major reservoir hosts (*1**,**4*). However, rodents, especially commensal rodents, may represent an additional HEV reservoir and may play a role in the epidemiology of hepatitis E. HEV-reactive antibodies have been detected in several rat species (*Rattus norvegicus*, *R. rattus, R. exulans)* but also in some noncommensal wild rodent species (*5**–**8*). By using broad-spectrum, nested, reverse transcription–PCR (RT-PCR), we recently detected HEV-like sequences in fecal samples of Norway rats (*R. norvegicus*) trapped as part of the Rodent-borne Pathogens network (which coordinates activities with regard to rodent trapping during outbreaks) (*9**,**10*). These sequence fragments had high nucleotide sequence divergence to genotypes 1–4 and to avian HEV strains.

## The Study

**Figure 1:**
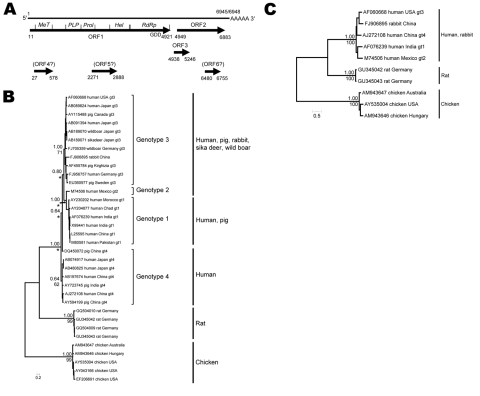
Genome structure and localization of putative open reading frames (ORFs) and functional domains in ORF1 of hepatitis E virus (HEV) sequences from Norway rats nos. 63 and 68, collected in Germany, July 2009 (A); phylogenetic trees based on a partial nucleotide sequence of 1,576 nt (B); and the complete genomes (C). RNA was isolated from liver samples by using the RNeasy Mini Kit and a QIAshredder (QIAGEN, Hilden, Germany). The entire rat HEV genome sequences of each rat were determined by a primer walking strategy and rapid amplification of cDNA ends protocols (GenBank accession nos. GU345042 and GU345043). ORFs were predicted by using the SeqBuilder Module of the DNASTAR software package (Lasergene, Madison, WI, USA). Putative functional domains in ORF1 were compared with those predicted in the corresponding regions of ORF1 from HEV genotypes 1–4 (*11*). The methyltransferase (MeT), helicase (Hel), and RNA-dependent RNA polymerase (RdRp, GDD motif indicated) domains are conserved and in the same order in the rat HEV genomes. In contrast, the papain-like protease domain (PLP) and the proline-rich domain (Prol) were more variable. Three additional ORFs (4, 5, 6) were predicted for both rat HEV genomes, for which no similar amino acid sequence could be found by BLASTp (http://blast.ncbi.nlm.nih.gov/Blast.cgi) search, sequence profile search in Pfam, and no functional pattern by Prosite (www.expasy.ch/prosite/) search; however, several similar sequences were retrieved from the Uniprot collection by comparison of translated nucleotide sequences with BLASTx (http://blast.ncbi.nlm.nih.gov/Blast.cgi). In addition, these ORFs showed less difference to the host codon usage than ORF3 as determined by Graphical Codon Usage Analyzer (http://gcua.schoedl.de/) and STRAP (http://3d-alignment.eu/). Phylogenetic relationships were reconstructed by using neighbor-joining and Bayesian algorithms after substitution model estimation (*12*). Robustness of nodes in phylogenetic trees is given above branches for Bayesian algorithms (sampling every 10 of 1 million generations; first 25,000 samples discarded as burn-in) and below branches for neighbor joining (10,000 bootstrap replicates). Only support values for main nodes that connect genotypes or major evolutionary lineages are displayed. *Indicates that neighbor-joining algorithms suggest instead a closer phylogenetic relationship between genotypes 3 and 4 with genotype 1 basal to these 2. Scale bar indicates phylogenetic distances in nucleotide substitutions per site.

During July 8–16, 2009, a total of 6 Norway rats, 3 male and 3 female, 65–432 g, were trapped in manholes of the sewer system of Hamburg, northern Germany, at the same locations where ≈12 months before HEV RNA had been detected in rat feces (*10*). Standardized necropsy (*9*) found no morphologic abnormalities. Initial serologic screening with a commercial genotype 1–based ELISA (Axiom, Bürstadt, Germany) detected no reactive antibodies in transudates of any of the 6 rats. Liver RNA from 1 female (no. 68, 311 g) and 1 male (no. 63, 313 g) rat yielded an amplification product of the expected size (331–334 nt) and a sequence identity of 83.8%–94.6 % with the HEV sequences recently obtained from rat feces (data not shown). Using a strategy according to Schielke et al. (*4*), we determined the entire rat HEV genome sequences from each sample to be 6,945 nt and 6,948 nt; the sequences differed by an insertion–deletion polymorphism in the 3′ noncoding region. The sequence identity between each complete sequence was 95.3% and reached 55.1%–55.9% to HEV genotypes 1–4 and 49.3%–50.2% to avian HEV strains (Table). Using prediction software, we identified the major ORFs 1, 2, and 3 in the new genomes in an organization typical for HEV (Figure 1, panel A). In contrast to HEV genotypes 1–3, rat HEV ORFs 1 and 3 do not overlap. Three additional putative ORFs of 280–600 nt that overlap with ORFs 1 or 2 were predicted for each rat HEV genome (Figure 1, panel A). However, before the meaning of these findings can be verified, sequence information from additional rat HEV strains and experimental proof are needed. Phylogenetic analyses of a 1,576-nt segment available for all published rat HEV sequences demonstrated clear separation from mammalian genotypes 1–4 and avian strains (Figure 1, panel B). The same 3 phylogenetic clusters were obtained when the complete genomes were analyzed (Figure 1, panel C) and when the nucleotide and deduced amino acid sequences of ORF1, ORF2, and ORF3 were investigated separately (data not shown).

**Table:** Nucleotide and deduced amino acid sequence identities between human, rabbit, and avian HEV strains compared with HEV isolated from 2 Norway rats, Germany, July 2009*

Strain, GenBank accession no.	Rat no., GenBank accession no.								
	63, GU345042					68, GU345043			
	Genome, nt	ORF1, aa	ORF2, aa	ORF3, aa		Genome, nt	ORF1, aa	ORF2, aa	ORF3, aa
Genotype 1, F076239	55.9	47.6	56.2	27.5		55.7	47.4	56.4	30.4
Genotype 2, M74506	55.3	48.7	55.4	28.4		55.2	48.6	55.5	29.4
Genotype 3, F060668	55.7	48.0	57.2	24.8		55.7	47.7	57.3	26.7
Genotype 4, J272108	55.5	48.2	55.9	27.5		55.3	47.8	56.1	26.5
Rabbit HEV, J906895	55.1	48.7	56.7	23.5		55.1	48.6	56.8	25.5
Avian HEV/Hungary, AM943646	50.2	46.5	45.9	26.9		49.9	46.4	46.3	26.9
Avian HEV/Australia, AM943647	49.9	46.6	46.1	26.9		49.3	46.5	46.5	26.9
Avian HEV/USA, AY535004	49.5	46.7	46.1	26.9		49.8	46.7	46.5	26.9

*HEV, hepatitis E virus; ORF, open reading frame.

**Figure 2:**
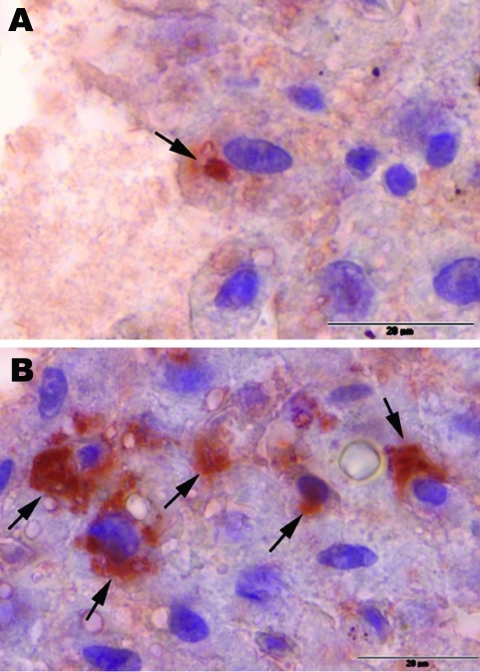
Immunohistochemical staining (peroxidase-antiperoxidase (PAP) technique) of liver samples from 2 rat-hepatitis E virus (HEV)–positive Norway rats from Germany, July 2009. Arrows indicate immunohistochemical positive reactions in the cytoplasm of single hepatocytes (A) and in a few foci in hepatocytes and stellate cells (B). For PAP staining, deparaffinized slides of liver samples were incubated with anti-HEV–positive human serum, which had been previously used to detect rat HEV by using solid phase immunoelectron microscopy (*10*), for 1 h at 37°C with protein A (Sigma-Aldrich, Steinheim, Germany) at a dilution of 1:100 for 45 min at 37°C and finally with PAP complexes from rabbits (Sigma, St. Louis, MO, USA) at a dilution 1:200 for 45 min at 37°C. AEC (3-amino-9-ethylcarbazol; Sigma Chemie GmbH, Deisenhofen, Germany) was used as the substrate chromogene. The slides were counterstained with hematoxylin and subsequently analyzed by light microscopy. Scale bars = 20 µm.

**Figure A1:**
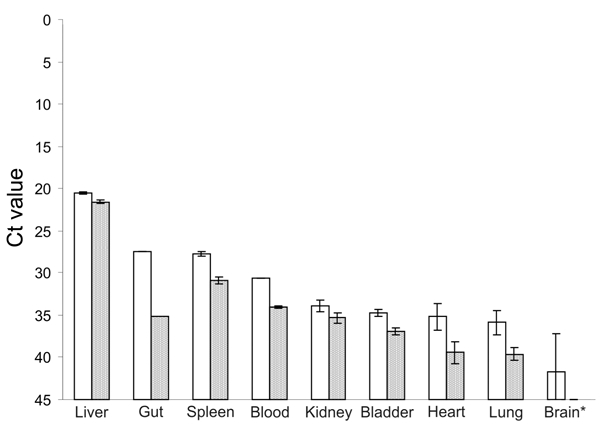
Results of real-time reverse transcription–PCR showing cycle threshold (Ct) values of viral load in different tissues of Norway rats nos. 63 (white bars) and 68 (gray bars). For RNA isolation, 10 mg of tissue or 10 µL of blood were homogenized and used. The PCR was selective for a region in the open reading frame 2 of rat hepatitis E virus (rHEV) and was based on primers rHEV-forward (5′-TACCCGATGCCGGGCAGT-3′) and rHEV-reverse (5′-ATCCACATCTGGGACAGG-3′) and probe (5′-6FAM-AATGACAGCACAGGCACC-BBQ-3′). Error bars indicate SD. *Brain sample from rat no. 63 contained blood.

To compare viral load in different tissues of the 2 HEV–positive rats, we developed a real-time RT-PCR selective for a region in the ORF2 of rat HEV. Parallel analysis of RNA isolated from 10 mg of each tissue or 10 µL of blood reproducibly showed the highest viral load to be in the liver; cycle threshold values for liver were 20.5 and 21.6 for each animal and lower for all other tissues (Figure A1). Further, immunohistochemical analysis, using anti-HEV serum, detected viral antigen in the cytoplasm of a few hepatocytes from each HEV-positive rat. Antigen was also observed in some activated hepatic stellate cells (Figure 2). Hematoxylin-eosin staining showed a marginally increased number of monocytes and granulocytes in sinusoids as well as a moderately increased number of lymphocytes and plasma cells in some Glisson triads of the livers (data not shown).

## Conclusions

Phylogenetic analyses and nucleotide and amino acid sequence comparisons demonstrated that the complete rat HEV genome sequences were consistently well separated from those of mammalian genotypes 1–4 and the tentative avian genotype. This finding suggests that these sequences represent an additional genotype (Figure 1, Table). In our analyses, the recently described HEV strain found in domestic rabbits, proposed to represent a separate genotype (*13*), clustered with human HEV genotypes irrespective of the genome part, nucleotide, or deduced amino acid sequences analyzed (Figure 1, panels B, C, and data not shown). Therefore, this strain may represent the consequence of recent spillover rather than the result of long-term virus–host coevolution. In contrast, the nonzoonotic avian HEV strains strongly differ from the mammalian HEV genotypes 1–4 (Figure 1, panels B, C). Although in the genus *Hepevirus* no species demarcation criteria have been defined, the marked sequence diversities suggest that the rat HEV represents an additional virus species other than HEV-1, HEV-2, HEV-3, HEV-4, and the tentative species avian HEV, which are currently classified in this genus (*14*).

Detection of rat HEV RNA and antigen in the liver cells of the infected Norway rats may indicate hepatotropism of this virus. Therefore, regarding its organ and cell-type tropism, this virus seems to be similar to the human and pig HEV genotypes (*15*). Because the virus was also detected in the intestine and, in the previous study, in feces (*10*), fecal–oral transmission as for genotypes 1–4 is plausible. The common properties of this virus and the human HEV genotypes suggest the usefulness of developing an HEV model in laboratory rats. In addition, the detection of rat HEV in animals from an urban region in Germany raises questions about the putative epidemiologic role of rat HEV for hepatitis E in humans.
